# Outcomes and patient satisfaction after pelvic organ prolapse surgery with and without mesh: a retrospective cohort study with prospective follow-up

**DOI:** 10.1007/s00404-026-08315-4

**Published:** 2026-03-06

**Authors:** Franziska Beer, Pia Schaufelberger, Thomas W. P. Friedl, Anna Lindner, Sabine Schütze, Wolfgang Janni, Miriam Deniz

**Affiliations:** University Hospital Ulm Clinic for Gynaecology and Obstetrics, Ulm, Germany

**Keywords:** Transvaginal mesh repair, Native tissue repair, Sacrocolpopexy, Freiburg index of patient satisfaction

## Abstract

**Purpose:**

This retrospective cohort study with prospective follow-up evaluates patient satisfaction and complication rates following pelvic organ prolapse (POP) surgery, comparing three surgical approaches: transvaginal native tissue repair, transvaginal mesh repair, and laparoscopic sacrocolpopexy.

**Methods:**

Long-term satisfaction and postoperative complications of patients who underwent POP surgery between 2014 and 2021 at the University Women’s Hospital of Ulm were assessed via structured telephone interviews. Patient satisfaction was evaluated using the validated Freiburg Index of Patient Satisfaction (FIPS) questionnaire.

**Results:**

Of 782 patients with POP surgery, 297 patients with primary surgeries at our clinic were included in the analysis. Patients of all three groups were highly satisfied according to FIPS with no significant difference (*p* = 0.058). Complication rate also did not differ significantly between the groups except for mesh erosions with 12.9% for transvaginal mesh repair and 2.0% for sacrocolpopexy (*p* = 0.027). Adverse events as pain, voiding difficulty, overactive bladder (OAB), and recurrence of POP requiring operative treatment had a significant impact on the satisfaction rate of patients.

**Conclusion:**

Patients of all three surgical approaches reported high satisfaction following POP surgery. Complication rate did not differ significantly except for mesh erosions. However, mesh erosions did not significantly impact patient satisfaction.

**Trail registration:**

The trial was registered in the German Clinical Trials Register (DRKS00031971) on 1 June 2023.

## What does this study add to the clinical work?

**Table:** 

This retrospective cohort study with prospective follow-up evaluates complication rate and satisfaction with pelvic organ prolapse surgery, depending on the surgical method (transvaginal native tissue versus mesh repair versus sacrocolpopexy) using the validated Freiburg index of patient satisfaction (FIPS). Satisfaction rate was high among all three groups with no significant difference. Complication rate also did not differ significantly between groups except for mesh erosions.	

## Introduction

Pelvic organ prolapse (POP) is a common condition, with a prevalence from 40 to 60% among parous women [1]. There is ongoing debate regarding the safety of synthetic mesh implants for POP repair. Glazener et al. reported mesh-related complications in 12% of women in the randomized controlled PROSPECT trial [2]. Conversely, the use of transvaginal mesh may be justified by lower recurrence rates. A 2017 Cochrane Review reported that permanent mesh for anterior compartment repair reduces recurrent anterior wall prolapse and the need for repeat surgery compared with native tissue repair [1]. A meta-analysis cited in the German–Austrian–Swiss guideline found that the risk of anterior vaginal wall recurrence is three times higher after native tissue repair compared to mesh repair [3]. A 2019 FIGO review summarized 24 national recommendations regarding mesh use for POP and stress urinary incontinence (SUI), concluding that vaginal mesh should be reserved for recurrent POP or patients at high risk of recurrence, whereas no restrictions apply to transabdominal mesh during sacrocolpopexy [4].

The use of vaginal mesh varies markedly worldwide, ranging from routine application to extreme caution [4]. On average, five years pass between approval of a transvaginal mesh device and publication of the first randomized clinical trial. In 2008, the U.S. Food and Drug Administration (FDA) issued a safety communication, later updated in 2011, highlighting serious complications associated with transvaginal mesh implants [4]. In 2016, the risk classification was raised from class II to class III, prompting manufacturers to cease production of posterior vaginal meshes; in 2019, the FDA issued a complete ban on meshes for anterior vaginal repair [6]. In Europe, the Scientific Committee on Emerging and Newly Identified Health Risks (SCENIHR) issued recommendations in 2015 emphasizing, among other aspects, patient characteristics and surgeon experience when considering transvaginal mesh implantation [7].

This highlights the need for a national data registry to systematically document outcomes and complications of transvaginal mesh implants, enabling evidence-based evaluation [4]. At present, complication registries vary substantially in methodology, reporting mechanisms, and obligatory participation.

The primary endpoint of this retrospective cohort study with prospective follow-up was long-term patient satisfaction following POP surgery at the University Women’s Hospital Ulm between 2014 and 2021, comparing transvaginal native tissue repair, transvaginal mesh repair, and sacrocolpopexy. Secondary endpoints included complication rates and the type of complications associated with each surgical method.

## Material and methods

This study was approved by the local ethics committee and registered at the DRKS DRKS00031971.

First, all patients with a documented POP surgery (operation and procedure code 5–704) at the University Women’s Clinic Ulm between 2014 and 2021 were identified. Patients were initially contacted by telephone and informed about the study’s purpose and objectives. After obtaining verbal consent, written informed consent forms were mailed. Up to three attempts were made to establish telephone contact with each patient. Once signed informed consent was returned, patients were contacted again for the structured telephone interview. Demographic and clinical data were retrieved from surgical and clinical records of the institution.

To assess patient satisfaction, the validated Freiburg Index of Patient Satisfaction (FIPS) was used. This interdisciplinary questionnaire evaluates treatment-related satisfaction following interventional and surgical procedures. It includes five items rated on a 6-point Likert scale; the total score is divided by the number of items, resulting in a final score between 1.0 (excellent) and 6.0 (very poor) [8]. In addition to the FIPS, patients were asked about the occurrence and timing of complications, duration of symptoms, whether symptoms existed before surgery, and whether any interventions were required. To verify whether patients recalled the correct type of surgery, they were asked to select the procedure from a multiple-choice list (transvaginal anterior or posterior repair, laparoscopic surgery with hysterectomy and sacral fixation). Patients were also asked whether a mesh had been implanted.

Three groups were formed based on the surgical technique: transvaginal native tissue repair (group 1), transvaginal mesh repair (anterior, posterior or apical fixation; group 2) and sacrocolpopexy (group 3). The native tissue repair was performed through a classic anterior colporrhaphy and/or posterior colporrhaphy and/or sacrospinous fixation of the vaginal apex or the uterus. For anterior mesh repair, the Anterior Elevate® mesh kit system was used between 2014 and 2016. From 2017 to 2020, Calistar S or P® (Promedon) was used for anterior or posterior repair. The InGYNious® implant (A.M.I.) was used from 2018 to 2021. For cervical or apical fixation, Splentis® or BSC Mesh® (A.M.I.) were used. For sacrocolpopexy, TiLOOP® (PFM medical) was used until 2018, and DynaMesh® (Dahlhausen) thereafter. The applied mesh implants enable a tension-free elevation and correction of pelvic organ prolapse.

For analysis of postoperative complication rates (pain, voiding difficulty, obstipation, dyspareunia, SUI, and OAB), only symptoms not present before surgery and persisting longer than three months were considered. To avoid misclassification, patients who underwent simultaneous SUI surgery (tension-free vaginal tape or Bulkamid® injection) were excluded from the analyses involving voiding difficulty, SUI, or OAB (10 patients in Group 1, 3 in Group 2, 9 in Group 3).

To analyze the association between satisfaction and persistent postoperative symptoms, patients were categorized as **satisfied** or **dissatisfied** using a FIPS cut-off of 2.6 (FIPS < 2.6 = satisfied; FIPS ≥ 2.6 = dissatisfied). This cut-off was defined by the study team as no validated threshold exists. All reported postoperative symptoms were included in this analysis, regardless of prior symptom history or symptom duration.

### Statistical analysis

Categorical variables are described using absolute and relative frequencies, while metric variables are described with mean, median, interquartile and range. Comparisons among groups defined by the type of surgery were conducted with the Kruskal–Wallis H test or the Mann–Whitney U test for the metric variables age, BMI, and FIPS score. Comparisons among groups for categorical variables were performed using the chi-square test as were tests for associations between patient satisfaction (FIPS score < 2.6/ ≥ 2.6) and symptoms/complications present after surgery. To evaluate whether the frequency of symptoms/complications changed following surgery, we used the McNemar test for significance of changes. Statistical analysis was performed with the statistical package IBM SPSS Statistics for Windows, Version 29 (IBM Corp., Armonk, N.Y., USA), a two-sided significance level of α = 0.05 was used throughout and there was no adjustment of the significance level for multiple comparisons.

## Results

**Fig. 1:**
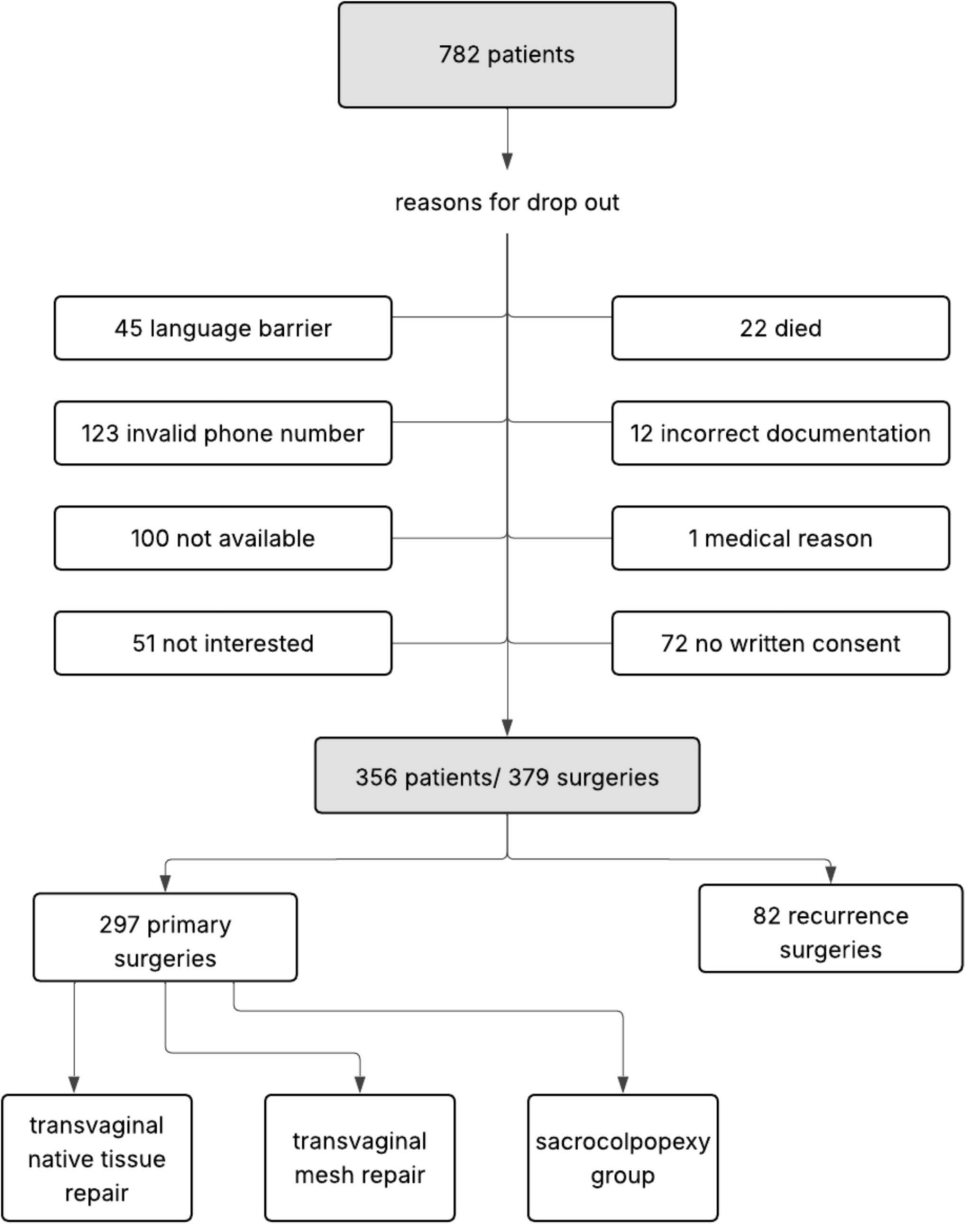
Patient flow diagram

Of the initially identified 782 patients with documented POP surgery at the University Women’s Clinic Ulm between 2014 and 2021, a substantial number had to be excluded for various reasons (Fig. 1). Completed questionnaires were obtained from 356 patients encompassing 379 surgeries (23 patients underwent both primary and recurrent procedures at our institution). After excluding 82 recurrent surgeries, the final dataset comprised 297 patients who underwent a primary POP surgery.

### Study population

Table 1 summarizes demographic and clinical characteristics, including POP-related symptoms at the time of primary surgery, across the three study groups. Significant differences were observed with respect to age (younger women in Group 3), incidence of hemorrhagic diathesis, cystocele, enterocele (all least frequent in Group 3), and uterine prolapse (descensus uteri), which was present in all women in Group 3. Voiding difficulty was most frequent in Group 2.

**Table 1:** Demographics and clinical characteristics before surgery of group 1 (transvaginal native tissue repair), group 2 (transvaginal mesh repair) and group 3 (sacrocolpopexy)

	Transvaginal native tissue repair Group 1 *n* = 165	Transvaginal mesh repair Group 2 *n* = 31	Sacrocolpopexy Group 3 *n* = 101	*p* value*
Demographics				
Age in years (median, range	67, 24–89	68, 46–87	58, 27–78	***p* < 0.001**
BMI in kg/m^2^ (median, range)	26.4, 17.4–41.3	26.6, 17.6–39.1	25.3, 19.5–36.9	*p* = 0.151
Nicotine	7.3% (*n* = 12)	3.2% (*n* = 1)	8.9% (*n* = 9)	*p* = 0.569
Diabetes mellitus	10.9% (*n* = 18)	6.5% (*n* = 2)	4.0% (*n* = 4)	*p* = 0.123
Hemorrhagic diathesis	17.0% (*n* = 28)	29.0% (*n* = 9)	7.9% (*n* = 8)	***p* = 0.048**
Clinical characteristics at the time of primary surgery				
Descensus uteri	85.5% (*n* = 141)	93.5% (*n* = 29)	100% (*n* = 101)	***p* < 0.001**
Cystocele	90.3% (*n* = 149)	96.8% (*n* = 30)	82.2% (*n* = 83)	***p* = 0.04**
Rectocele	89.7% (*n* = 148)	90.3% (*n* = 28)	86.1% (*n* = 87)	*p* = 0.641
Enterocele	4.2% (*n* = 7)	9.7% (*n* = 3)	0% (*n* = 0)	***p* = 0.021**
Stress urinary incontinence (SUI)	49.7% (*n* = 82)	61.3% (*n* = 19)	51.5% (*n* = 52)	*p* = 0.495
Overactive Bladder (OAB)	50.9% (*n* = 84)	51.6% (*n* = 16)	43.6% (*n* = 44)	*p* = 0.475
Voiding difficulty	17.6% (*n* = 29)	38.7% (*n* = 12)	16.8% (*n* = 17)	***p* = 0.017**
Obstipation	20.6% (*n* = 34)	19.4% (*n* = 6)	16.8% (*n* = 17)	*p* = 0.750
Dyspareunia	6.7% (*n* = 11)	6.5% (*n* = 2)	13.9% (*n* = 14)	*p* = 0.121

^*^Kruskal–Wallis H test for age and BMI, chi-squared test for all other parameters

### Patient’s satisfaction

**Fig. 2:**
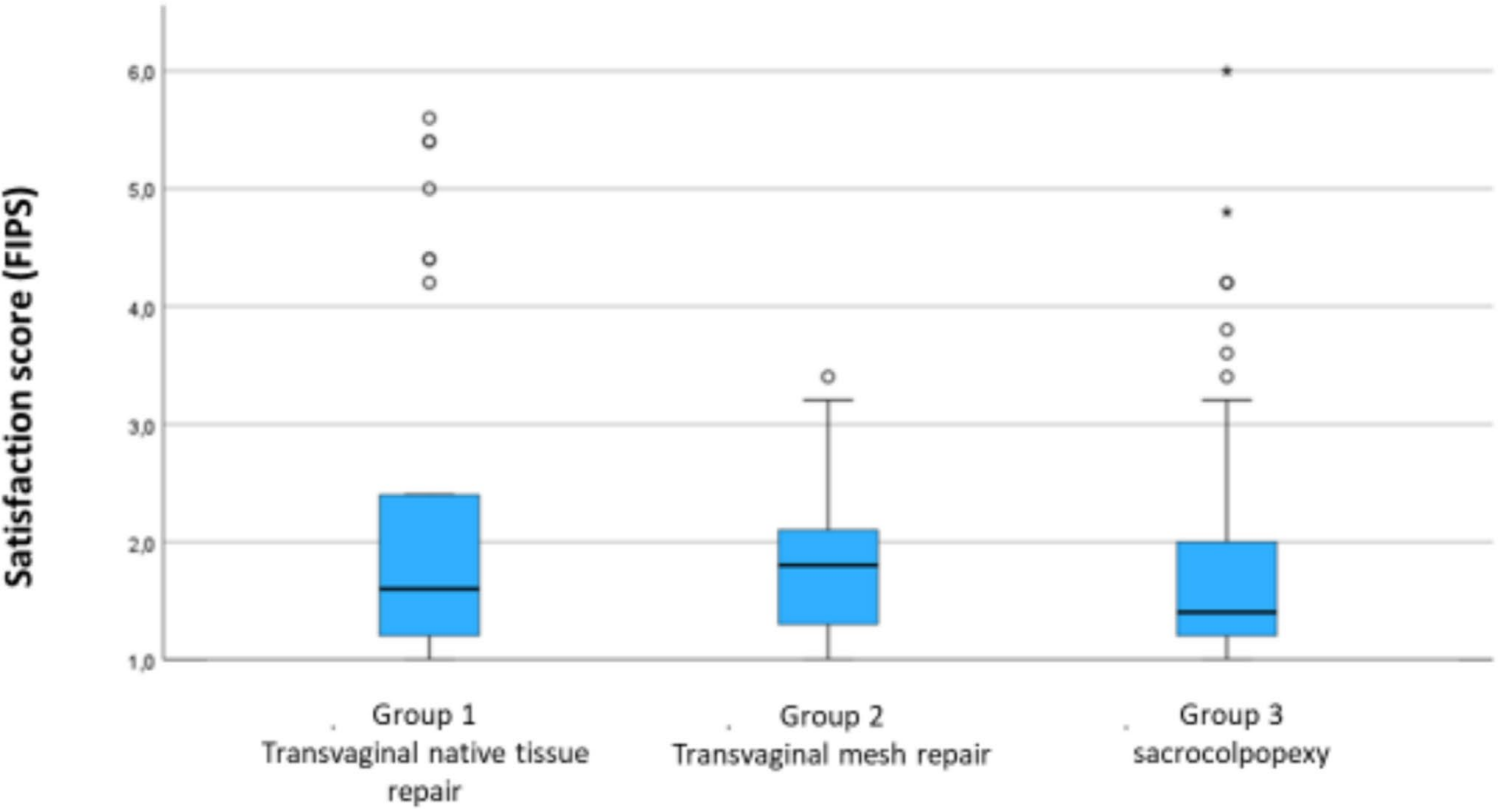
Satisfaction score according to Freiburg index of patient satisfaction (FIPS) of group 1 (transvaginal native tissue repair), group 2 (transvaginal mesh repair) and group 3 (sacrocolpopexy). Lower scores indicate higher satisfaction

The average FIPS score for the entire cohort was 1.90 (median 1.6, interquartile 1.2–2.2, range 1.0–6.0). Patients in group 3 reported the highest satisfaction (mean 1.76, median 1.4, interquartile 1.2–2.0, range 1.0–6.0), followed by women of group 2 (mean 1.83, median 1.8, interquartile 1.2–2.2, range 1.0–3.4) and women of group 1 (mean 2.0, median 1.6, interquartile 1.2–2.4, range 1.0–5.6). However, these differences did not reach statistical significance although the p value approached the significance threshold (*P* = 0.058; see also Fig. 2).

### Postoperative complication rates

No significant differences were observed in postoperative complication rates among the three groups, except for mesh erosions, which were significantly more common in Group 2 than in Group 3 (Table 2). Among the six cases of mesh erosion, all were managed by partial mesh excision. Two cases (both in Group 2) were managed in an outpatient setting, while three required operative excision (one in Group 2, two in Group 3). No complete mesh removal was necessary. One patient in Group 2 reported a mesh erosion in the questionnaire that was not confirmed in clinical records; instead, the patient had intermittent pain and voiding difficulties related to the mesh.

**Table 2:** Complication rate

	Transvaginal native tissue repair Group 1 *n* = 165	Transvaginal mesh repair Group 2 *n* = 31	Abdominal or laparoscopic sacrocolpopexy Group 3 *n* = 101	*p* value*
Hematoma	7.3% (*n* = 12)	9,7% (*n* = 3)	4.0% (*n* = 4)	*p* = 0.431
Pain	5.5% (*n* = 9)	3.2% (*n* = 1)	12.9% (*n* = 13)	*p* = 0.056
Voiding difficulty	1.9% (*n* = 3)	0% (*n* = 0)	2.1% (*n* = 1)	*p* = 0.687
Obstipation	3.6% (*n* = 6)	3.2% (*n* = 1)	9.9% (*n* = 10)	*p* = 0.084
Dyspareunia	3.0% (*n* = 5)	3.2% (*n* = 1)	5.9% (*n* = 6)	*p* = 0.490
Stress urinary incontinence (SUI)	12.3% (*n* = 19)	10.7% (*n* = 3)	13.0% (*n* = 12)	*p* = 0.946
Overactive bladder (OAB)	12.9% (*n* = 20)	14.3% (*n* = 4)	7.6% (*n* = 7)	*p* = 0.387
Mesh erosion		12.9% (*n* = 4)	2.0% (*n* = 2)	***p* = 0.027**
Subjectively perceived recurrence	45.5% (*n* = 75)	45.2% (*n* = 14)	42.6% (*n* = 43)	*p* = 0.897
Recurrence requiring conservative treatment	8.5% (*n* = 14)	6.5% (*n* = 2)	3.0% (*n* = 3)	*p* = 0.204
Recurrence requiring operative treatment	9.7% (*n* = 16)	9.7% (*n* = 3)	11.9% (*n* = 12)	*p* = 0.843

Pain, voiding difficulty, obstipation, dyspareunia, stress urinary incontinence (SUI) and overactive bladder (OAB) that did not exist before the surgery and persisted longer than 3 months. Group 1 (transvaginal native tissue repair), group 2 (transvaginal mesh repair) and group 3 (sacrocolpopexy). Please note that for the calculation of the complications voiding difficulty, SUI and OAB, patients were excluded who had simultaneous surgery for SUI (see Methods)

*chi-squared test

19 patients reported a hematoma after surgery; however, 13 of these cases were not documented clinically. 11 of these patients reported that the hematoma resolved within two weeks, and two within two months. One patient in Group 2 experienced an intraoperative bleeding requiring suturing and also reported persistent hypermenorrhea postoperatively. Four clinically documented hematomas were treated conservatively. One patient in Group 1 who had undergone concurrent vaginal hysterectomy developed vault bleeding requiring operative therapy two weeks later.

Of the 23 patients who reported postoperative pain, 3 had no pain documented at follow-up, and another 3 did not attend postoperative follow-up visits. Ten patients reported abdominal and/or back pain, four reported pelvic floor pain, and three reported pain associated with POP recurrence.

Nearly half of the patients reported a subjective recurrence; however, recurrence requiring surgical treatment occurred in only 9.7%, 9.7%, and 11.9% of patients in Groups 1, 2, and 3, respectively.

Overall, 76 complications required operative intervention (Table 3), with most procedures (*n* = 63) performed at our clinic.

**Table 3:** Complications that needed an operative treatment, distinguishing between treatment in our clinic and in an external clinic

	In our clinic	External clinic
Hematoma (*n*)	2	0
Pain (*n*)	5	0
Voiding difficulty (*n*)	6	0
Obstipation (*n*)	1	0
Dyspareunia (*n*)	0	0
Stress urinary incontinence (SUI) (*n*)	19	4
Overactive bladder (*n*)	4	1
Mesh erosion (*n*)	3	0
recurrence (*n*)	23	8
Total (*n*)	**63**	**13**

### Changes in symptoms following POP surgery

Table 4 presents symptom presence before and after surgery. Obstipation, dyspareunia, and voiding difficulty more frequently developed after surgery than resolved, with changes in obstipation and voiding difficulty reaching statistical significance. In contrast, SUI and OAB more often resolved after surgery than newly developed; however, these changes did not reach statistical significance.

**Table 4:** Presence of symptoms before and after surgery

	Obstipation	Dyspareunia	Voiding difficulty	Stress urinary incontinence	Overactive bladder
Symptoms absent before and after surgery	68.7% (*n* = 204)	83.2% (*n* = 247)	60.4% (*n* = 166)	39.3% (*n* = 108)	41.1% (*n* = 113)
Symptoms appeared after surgery	12.1% (*n* = 36)	7.7% (*n* = 23)	18.9% (*n* = 52)	13.1% (*n* = 36)	12.4% (*n* = 34)
Symptoms disappeared after surgery	2.7% (*n* = 8)	4.0% (*n* = 12)	11.3% (*n* = 31)	17.5% (*n* = 48)	14.9% (*n* = 41)
Symptoms present before and after surgery	16.5% (*n* = 49)	5.1% (*n* = 15)	9.5% (*n* = 26)	30.2% (*n* = 83)	31.6% (*n* = 87)
*p* value*	*p*** < 0.001**	*p* = 0.091	***p* = 0.028**	*p* = 0.230	*p* = 0.488

To avoid misinterpretation of the rates of voiding difficulty, SUI and OAB, patients who also had an operative therapy for SUI (tension-free vaginal tape or Bulkamid injection; *n* = 22) were excluded (see Methods)

*McNemar test for significance of changes

### Association between satisfaction and symptoms/complications after surgery

Patients with lower satisfaction (FIPS ≥ 2.6) more frequently experienced pain, voiding difficulties, OAB, subjective recurrence, and recurrence requiring surgical treatment compared with satisfied patients (FIPS < 2.6) (Table 5).

**Table 5:** Association between satisfaction according to Freiburg index of patient satisfaction (FIPS) and symptoms/complications after POP surgery

Symptom/complication	FIPS < 2.6 *n* = 242	FIPS ≥ 2.6 *n* = 55	*p* value*
Hematoma	5.4% (*n* = 13)	10.9% (*n* = 6)	*p* = 0.134
Pain	14.9% (*n* = 36)	40.0% (*n* = 22)	***p* < 0.001**
Voiding difficulty	26.0% *n* = 63	41.8% *n* = 23	***p* = 0.020**
Obstipation	28.5% (*n* = 69)	29.1% (*n* = 16)	*p* = 0.932
Dyspareunia	12.0% (*n* = 29)	16.4% (*n* = 9)	*p* = 0.380
Stress urinary incontinence (SUI)	41.7% (*n* = 101)	52.7% (*n* = 29)	*p* = 0.138
Overactive Bladder (OAB)	40.9% (*n* = 99)	60.0% (*n* = 33)	***p* = 0.010**
Mesh erosion^§^	5.2% (*n* = 6)	0% (*n* = 0)	*p* = 1.000
Subjectively perceived recurrence	36.0% (*n* = 87)	81.8% (*n* = 45)	***p* < 0.001**
Recurrence requiring conservative treatment	5.8% (*n* = 14)	9.1% (*n* = 5)	*p* = 0.364
Recurrence requiring operative treatment	8.3% (*n* = 20)	20% (*n* = 11)	***p* = 0.010**

*chi-squared test; ^§^ patients in group 2 or 3 only

Patients who underwent mesh-based procedures were more likely to correctly recall the type of surgery than those who underwent native tissue repair. Only about half of the patients in Group 1 accurately identified their procedure (Table 6).

**Table 6:** Patients of group 1 (transvaginal native tissue repair), group 2 (transvaginal mesh repair) and group 3 (sacrocolpopexy) who were able to recall the method of their surgery in a multiple-choice question correctly

	Transvaginal native tissue repair Group 1 *n* = 165	Transvaginal mesh repair Group 2 *n* = 31	sacrocolpopexy Group3 *n* = 101	*p* value
Stated surgery method correctly	49.1% *n* = 81	74.2% *n* = 23	80.2% *n* = 81	*p* < 0.001

## Discussion

Pelvic organ prolapse is common with 14–19% of women undergoing a surgical correction during their lifetime [9]. The aims of this study were to evaluate long-term patient satisfaction and complication rates following POP surgery, with particular focus on comparing sacrocolpopexy, transvaginal mesh repair, and native tissue repair. While sacrocolpopexy is well established and widely accepted, the use of transvaginal mesh remains controversial [4, 10, 11]. Most existing studies examine either sacrocolpopexy or transvaginal mesh repair in isolation. Therefore, the present discussion primarily compares our findings with studies involving transvaginal mesh versus native tissue repair.

### Recurrence and adverse events

Use of vaginal mesh is generally reserved for patients at high risk of recurrence or with recurrent POP [4]. According to a Cochrane review by Maher et al., the recurrence risk of anterior vaginal wall prolapse is 13% after mesh repair versus 32%–45% after native tissue repair, with reoperation rates of 2% for mesh and 2%–7% for native tissue repair; most included RCTs had follow-up durations of only 2–3 years [1]. In our study, no significant differences were observed in subjectively perceived recurrence rates or recurrences requiring conservative or operative treatment, noting that recurrence assessment relied on patient self-report (Table 2). The multi-center randomized controlled trial PROSPECT including 865 women, 435 undergoing transvaginal mesh repair and 430 transvaginal standard repair, evaluated the outcomes of prolapse repair with a follow-up of 2 years, also analyzing the occurrence of symptomatic POP. No significant difference was found in symptomatic prolapse (82% standard versus 85% mesh repair *p* < 0.3) using the Pelvic Organ Prolapse Symptom Score (POP-SS) [2]. In accordance with the PROSPECT study, we also observed no significant difference regarding subjectively perceived recurrences following native tissue or mesh repair. However, the rates observed in our study are considerably lower (45.5% for native tissue repair, 45.2% for mesh repair), which might at least partly be due to the non-validated questionnaire, that was used for this analysis. Altman et al. published the results of a multi-center randomized controlled trial comprising 200 patients with transvaginal mesh repair and 189 patients with traditional colporrhaphy of the anterior vaginal wall with a follow-up of one year, reporting recurrence surgery rates of 0.5% in the traditional colporrhaphy group and 0% in the mesh group [12]. In comparison, the rate of operative treatment for recurrence of POP in our cohort was higher with 9.7% for both vaginal mesh and native tissue repair, likely due to the substantially longer follow-up (2–9 years). The recurrence rate may be overestimated due to the high drop-out rate, as women with intervention-requiring recurrences were more likely to participate in the study.

In addition to the rationale for the use of transvaginal mesh implants in the prevention of recurrence, the potential risk of adverse events must also be carefully considered. Mesh erosion and dyspareunia are the most frequently cited mesh-related complications. Vaginal mesh exposure rates of 3%–19% have been reported [12–15], consistent with our rate of 12.9% (*n* = 4) in the transvaginal mesh group. Notably, one patient reported mesh erosion that was not confirmed clinically—underlining the limitations of subjective reporting. De novo dyspareunia after surgery for POP occurs in 3–9% for transvaginal mesh repair and 5%–16% for native tissue repair with no significant difference between the groups [2, 16, 17]. This is in line with our data with a rate of de novo dyspareunia of 3.0% for native tissue and 3.2% for transvaginal mesh repair. Evaluation of perioperative bleeding complication is rare; when reported, the focus is usually on transfusion rates [16–18]. Postoperative hematoma is rarely analyzed. Altman et al. recorded vaginal wound bleeding between hospital discharge and 2 months after surgery with a rate of 0.5% in each group (mesh and native tissue repair) [12]. Rudnicki et al. recorded a rate of hematoma of 0% for native tissue and 1.2% for mesh repair during hospital stay [14]. In the present study, 7.3%, 9.7%, and 4.0% of patients reported hematoma after native tissue repair, mesh repair and sacrocolpopexy, respectively. However, out of the 19 patients, 13 patients did not have a hematoma according to our clinical records, suggesting that these patients might have had normal postoperative bloody discharge and misunderstood the question about a hematoma over the phone. Urinary incontinence, voiding difficulties, and obstipation showed no significant differences between surgical methods, consistent with previous studies [2, 14, 15, 17]. Postoperative pain is seldom analyzed in POP literature. In our study, postoperative pain significantly impacted patient satisfaction, underscoring the need for its systematic assessment. Altman et al. reported pelvic/genital pain rates of 0% (native tissue) and 0.5% (mesh) between 2 months and 1 year [12]. Although our pain rates were higher, no significant differences were observed between surgical approaches, although pain tended to be more frequent after sacrocolpopexy.

### Changes in symptoms after POP surgery

Ramanah et al., in a prospective study of 151 patients, reported significant postoperative decreases in voiding difficulty and SUI, and a non-significant decrease in urge incontinence [19]. In contrast, obstipation and voiding difficulty occurred significantly more often as new symptoms in our cohort. Nieminen et al. observed fewer patients with impaired rectal emptying three years after surgery than before, though no statistical testing was reported [15].

### Satisfaction

Especially in the context of urogynaecological symptoms, which are rarely life-threatening but can severely restrict quality of life, patient satisfaction is of high importance. High satisfaction according to FIPS was observed across all three surgical groups. These findings align with Carey et al., who reported high satisfaction (VAS ≥ 80/100) in both mesh (91.5%) and non-mesh groups (81.0%) [13]. Studies using the validated Prolapse Quality of Life questionnaire (P-QOL) similarly found no significant difference in postoperative QoL between native tissue repair and mesh repair [1]. Zhang et al. reported that, in an 11 year follow-up of transvaginal mesh implantation, 94.2% of patients achieved a Patient Global Impression of Improvement (PGI-I) score ≤ 2 (very much better or much better) [20]. Interestingly, pain, voiding difficulty, OAB, and recurrence requiring surgery significantly reduced satisfaction in our study, whereas mesh erosion and dyspareunia—two highly debated mesh-associated complications—did not.

### Limitation and strengths

Limitations include the retrospective design and reliance primarily on subjective patient reports. Nevertheless, prospective follow-up data were collected. Additionally, a high drop-out rate was observed, which complicates the interpretation of the results as it likely introduces a significant bias. While FIPS is validated for assessing patient satisfaction, the remaining questionnaire items were not standardized. Due to the retrospective nature, follow-up duration varied between 2 and 9 years; however, this is longer than most published RCTs, which typically report 1–3 years of follow-up. A major strength of this study is the comparatively large sample size and the inclusion of real-world data from routine clinical practice.

## Conclusion

This retrospective cohort study with prospective follow-up provides single-institution real-world data on outcomes after POP surgery. Overall satisfaction was high across all surgical groups, with no significant differences between transvaginal mesh repair, native tissue repair, and sacrocolpopexy. Recurrence and complication rates were comparable across surgical modalities, except for mesh erosion, which occurred more frequently after transvaginal mesh repair. However, mesh erosion did not significantly affect postoperative patient satisfaction. Pain, voiding difficulty, OAB, and recurrence requiring surgical intervention had the strongest negative impact on patient satisfaction. These findings underscore that individual patient characteristics and preferences remain essential factors in surgical decision-making. A national—or ideally international—complication registry would provide valuable real-world data on POP surgery, particularly mesh-related outcomes, and could help guide clinical decision-making beyond what is captured in randomized trials.

## Data Availability

The data that support the findings of this study are not openly available due to reasons of sensitivity and are available from the corresponding author upon reasonable request. Data are located in controlled access data storage at University Hospital Ulm.
